# Assessing Transcriptome Quality in Patch-Seq Datasets

**DOI:** 10.3389/fnmol.2018.00363

**Published:** 2018-10-08

**Authors:** Shreejoy J. Tripathy, Lilah Toker, Claire Bomkamp, B. Ogan Mancarci, Manuel Belmadani, Paul Pavlidis

**Affiliations:** ^1^Michael Smith Laboratories, University of British Columbia, Vancouver, BC, Canada; ^2^Department of Psychiatry, University of British Columbia, Vancouver, BC, Canada

**Keywords:** gene expression profiling, patch-clamp techniques, sequencing data analysis, neurophysiology, meta-analysis, ion channels, cell types

## Abstract

Patch-seq, combining patch-clamp electrophysiology with single-cell RNA-sequencing (scRNAseq), enables unprecedented access to a neuron's transcriptomic, electrophysiological, and morphological features. Here, we present a re-analysis of five patch-seq datasets, representing cells from *ex vivo* mouse brain slices and *in vitro* human stem-cell derived neurons. Our objective was to develop simple criteria to assess the quality of patch-seq derived single-cell transcriptomes. We evaluated patch-seq transcriptomes for the expression of marker genes of multiple cell types, benchmarking these against analogous profiles from cellular-dissociation based scRNAseq. We found an increased likelihood of off-target cell-type mRNA contamination in patch-seq cells from acute brain slices, likely due to the passage of the patch-pipette through the processes of adjacent cells. We also observed that patch-seq samples varied considerably in the amount of mRNA that could be extracted from each cell, strongly biasing the numbers of detectable genes. We developed a marker gene-based approach for scoring single-cell transcriptome quality *post-hoc*. Incorporating our quality metrics into downstream analyses improved the correspondence between gene expression and electrophysiological features. Our analysis suggests that technical confounds likely limit the interpretability of patch-seq based single-cell transcriptomes. However, we provide concrete recommendations for quality control steps that can be performed prior to costly RNA-sequencing to optimize the yield of high-quality samples.

## Introduction

Linking gene expression to a neuron's electrical and morphological features has long been a goal of cellular neuroscience. To this end, one strategy is to use the same patch-clamp electrode for electrophysiological characterization for mRNA sampling, for example, by aspirating the cell's cytosol into the patch-pipette (Eberwine et al., 1992; Sucher and Deitcher, 1995; Toledo-Rodriguez et al., 2004; Kodama et al., 2012; Rossier et al., 2014; Toledo-Rodriguez and Markram, 2014). The aspirated mRNA transcripts can then be detected and quantified using RT-PCR (Eberwine et al., 1992; Sucher and Deitcher, 1995; Cauli et al., 1997, 2000; Toledo-Rodriguez et al., 2004; Kodama et al., 2012; Pfeffer et al., 2013; Rossier et al., 2014) or other methods (Subkhankulova et al., 2010), allowing the quantification of multiple genes or transcripts.

Recently, a number of groups have published protocols for patch-seq that extend previous RT-PCR-based methods by quantifying patch-pipette sampled cellular mRNA transcripts using next-generation RNA-sequencing (Cadwell et al., 2015, 2017a,b; Bardy et al., 2016; Chen et al., 2016; Földy et al., 2016; Fuzik et al., 2016; Pfeffer and Beltramo, 2017; Muñoz-Manchado et al., 2018; van den Hurk et al., 2018). These protocols make use of recent technical improvements in single-cell RNA-sequencing (scRNAseq) that enable gene expression quantification from very low starting amounts of mRNA (Poulin et al., 2016; Tasic et al., 2017), such as those present in a single-cell or single-nucleus. Importantly, patch-seq is transcriptome-wide (in principle), in contrast to RT-PCR where PCR primers for each sampled transcript need to be explicitly specified (Toledo-Rodriguez and Markram, 2014).

Patch-seq mRNA sample collection differs from standard single-cell or single-nucleus RNAseq, in two major ways (Cadwell et al., 2017a,b). First, as opposed to relying on dissociating cells into suspension, the micropipette used for electrical recording is used for mRNA extraction via aspiration. While guiding the patch pipette to (or from) the soma of a cell of interest, the pipette often must travel through the processes of other cells, presenting an opportunity for contamination. Second, the effectiveness of cell content aspiration is difficult to control, so the amount of mRNA extracted may tend to vary from cell to cell.

Here, our goal was to investigate the quality of scRNAseq data profiled using patch-seq. Our strategy was to compare patch-seq derived scRNAseq data with analogous data sampled using cellular-dissociation based methods, from which multiple large and high-quality single-cell transcriptomic datasets are available (Zeisel et al., 2015; Tasic et al., 2016). Our findings suggest that sampling cellular mRNA using a patch-pipette induces technical artifacts that tend not to be present to the same degree in cellular-dissociation based scRNAseq data. Based on our findings, we provide approaches for detecting these technical issues and discuss strategies for generating high-quality patch-seq datasets in the future.

## Methods

### Dataset overview

We made use of five previously published patch-seq datasets (“Cadwell,” “Földy,” “Fuzik,” “Bardy,” “Chen”; Cadwell et al., 2015; Bardy et al., 2016; Chen et al., 2016; Földy et al., 2016; Fuzik et al., 2016). These datasets were identified using a PubMed keyword search for the term “patch-seq” or “patch seq,” reflecting, to our knowledge, all of the published patch-seq datasets as of October 2017. The Cadwell, Földy, and Fuzik datasets were collected from acute brain slices from adult and juvenile mice whereas the Bardy and Chen datasets were collected from human stem-cell derived neurons and astrocytes in culture. We did not analyze one patch-seq dataset (Pfeffer and Beltramo, 2017); GSE90822, because intrinsic electrophysiological characterization was not performed on the cells prior to mRNA harvest. We did not find patch-seq datasets collected from cultured or acutely dissociated mouse neurons. We chose not to re-analyze single-cell RT-PCR datasets, such as (Cauli et al., 2000; Toledo-Rodriguez et al., 2004; Rossier et al., 2014), as these typically do not quantify marker gene expression for non-neuronal cell types.

We compared the five patch-seq datasets to two cellular dissociation-based single-cell RNAseq datasets (Tasic, Zeisel; Zeisel et al., 2015; Tasic et al., 2016). We downloaded single-cell transcriptomic data from each study from accessions provided in Table 1 and Supplementary Table 1 or by contacting the authors directly. We obtained patch-seq-based electrophysiological data for the Cadwell and Fuzik datasets from the authors. For all patch-seq datasets, electrophysiological data were provided as a spreadsheet containing a set of summarized electrophysiological features per cell (e.g., input resistance, resting membrane potential, etc.). Electrophysiological data from the Allen Institute Cell Types database (celltypes.brain-map.org) were obtained and processed as described previously (Tripathy et al., 2017).

**Table 1:** Description of patch-seq datasets re-analyzed in this study.

**Dataset**	**Description**	**Preparation**	**RNA amplification**	**Number of cells**	**Accession**
Cadwell et al., 2015	Cortical layer 1 interneurons	Acute mouse slices	Smart-seq2	57	E-MTAB-4092
Fuzik et al., 2016	Cortical layer 1/2 interneurons and pyramidal cells	Acute mouse slices	STRT-C1 (with unique molecule identifiers)	80	GSE70844
Földy et al., 2016	Hippocampal CA1 and Subiculum pyramidal cells and regular- and fast-spiking interneurons	Acute mouse slices	SMARTer	93	GSE75386
Bardy et al., 2016	Stem-cell derived neurons and astrocytes	Differentiated human cells in culture	SMARTer	56	NA^*^
Chen et al., 2016	Stem-cell derived neurons	Differentiated human cells in culture	NEBNext Ultra DNA Library Prep Kit	20	GSE77564

* *Expression data obtained by contacting the authors directly*.

### Transcriptome data pre-processing

We reprocessed transcriptomic data for the Cadwell, Földy, and Tasic datasets directly from Gene Expression Omnibus (GEO) or Array Express. Data from GEO was downloaded using fastq-dump version 2.8.2 from the Sequence Read Archive Toolkit. Technical reads, such as barcodes and primers were filtered out during extraction. Adapter sequences were clipped from the raw reads. The list of option used is as follows: “–gzip –skip-technical –readids –dumpbase –split-files –clip.” Data from ArrayExpress was downloaded and used directly as prepared by the European Bioinformatics Institute.

The reference mouse transcriptome was produced using the “rsem-prepare-reference” script provided by the RNA-seq by expectation-maximization (RSEM) RNA-Seq transcript quantifier (Li and Dewey, 2011). The assembly version used was Ensembl GRCm38, packaged by Illumina for the iGenomes collection. Alignment was performed using STAR (Dobin et al., 2013) version 2.4.0h, provided as the aligner to RSEM v1.2.31. Default parameters were used (with the exception of parallel processing and logging related options). Transcript definitions used to detect external RNA controls consortium (ERCC) spike-ins were obtained from the ERCC92 version fasta and GTF files. Spike-ins were concatenated to the GRCm38 assembly before applying rsem-prepare-reference, and independently to create a standalone ERCC assembly. Both the concatenated and standalone spike-ins assemblies showed highly comparable proportions of spike-in expression. Reprocessed counts from the Cadwell, Földy, and Tasic were summarized as transcripts per million (TPM).

For the Fuzik and Zeisel datasets, we made use of the quantified summarized unique molecule counts (UMIs) made available at GEO. One advantage of UMIs is that they tag unique mRNA molecules prior to PCR and help control for biases in PCR amplification (Tasic et al., 2017). To account for differences in detected UMI counts per cell, we normalized the Fuzik and Zeisel datasets as UMI counts per million. For the Bardy and Chen human datasets, we used the summarized count matrices directly provided by the authors, provided as transcripts per million (TPM; Bardy) or fragments per kilobase per million (FPKM; Chen).

### Mapping of mouse patch-seq cell types onto taxonomies derived from dissociated cells

Using descriptions for cellular identities provided in the original patch-seq publications, we manually mapped each of the cell types represented across the three mouse patch-seq datasets onto transcriptomically-defined cellular clusters reported in the two dissociated cell datasets (shown in Supplementary Table 2). For example, given that the elongated neurogliaform cells and single bouquet cells characterized by Cadwell et al. (2015) are both cortical layer 1 cells, we manually mapped these to the layer 1 cells defined in Tasic as Ndnf cells. Similarly, we mapped the hippocampal regular-spiking interneurons characterized in Foldy to the Sncg cluster from Tasic (personal communication with Csaba Földy). To align cell subtype clusters between Tasic and Zeisel, we used mappings provided by MetaNeighbor (Crow et al., 2018; shown in Supplementary Table 2). The mappings between broad cell types in Tasic with Zeisel are provided in Supplementary Table 3. We note that there is some ambiguity in our mapping of analogous cell types across datasets; ideally, such cross-dataset cell type mappings would be guided by the use of transgenic mouse lines with specific cell types labeled by florescent proteins (Madisen et al., 2010; Pfeffer and Beltramo, 2017).

### Identification of cell type-specific marker genes

For this study, we defined two classes of marker genes, termed “on” and “off” markers. The first class, “on” markers, are genes that are highly and ubiquitously expressed in the cell type of interest with enriched expression relative to other cell types. The second class, “off” markers, are expected to be expressed at low levels in a given patch-seq cell type. These are genes that are specifically expressed in a single cell type (e.g., microglia) and, if expressed in combination with markers specific to other cell types, are an indicator of possible cellular contamination. To identify marker genes, we employed two recent surveys of mouse cortical diversity from Tasic et al. and Zeisel et al. (Zeisel et al., 2015; Tasic et al., 2016).

To identify “on” marker genes, we initially used the Tasic dataset, and selected genes whose average expression in the chosen cell type was >10 times relative all other cell types in the dataset, with an average expression in the cell type of >100 transcripts per million (TPM). From this initial gene list, we next filtered genes expressed at >10 TPM/cell in >75% of all cells of that type in Tasic, and >1 UMI/cell in >50% of all cells of that type in Zeisel. Using the Tasic nomenclature, we defined “on” markers for Ndnf, Sncg, Pvalb, and Pyramidal cell types.

To identify “off” marker genes for broad cell types (shown in Supplementary Table 3), as an initial listing we used the set of cell type-specific marker genes for broad cell classes in the mouse cortex, defined in our previous work using the NeuroExpresso database (Mancarci et al., 2017). Specifically, we used the set of cortical markers derived from single-cell RNA-seq for astrocytes, endothelial cells, microglia, oligodendrocytes, oligodendrocyte precursor cells, and pyramidal cells. From this list, we first filtered out lowly expressed genes that were expressed <10 TPM/cell in >50% of all cells of that type in Tasic, and <1 UMI/cell in >50% of all cells of that type in Zeisel. Next, we filtered genes that were moderately expressed in our patch-seq cell types of interest by assessing the expression of these genes in the Ndnf, Sncg, Pvalb, and Pyramidal cell types, removing genes that were expressed at a level >10 TPM/cell in >33% of all cells of that type in Tasic, and >2 UMI/cell in >33% of all cells of that type in Zeisel.

When defining “on” and “off” marker genes for inhibitory cell subtypes (e.g., the Ndnf cell type), we did not compare these cells to other GABAergic cells. For example, when defining “on” markers for Ndnf cells, we did not compare these cells' expression to Pvalb or Sst cells. We note that this choice limits our ability to identify inhibitory-to-inhibitory cell contamination, for example, an Ndnf cell contaminated by Sst-cell specific markers. To define an initial set of “off” markers for GABAergic inhibitory cells, we first obtained a list of genes based on Tasic where in GABAergic cells had average expression >10 times all other non-GABAergic cells in the dataset and with an average expression of at least 100 TPM.

The final list of filtered mouse cell type specific marker genes used in this study are provided in Supplementary Table 4.

To obtain a list of human cell type specific marker genes for use for the Bardy and Chen datasets, we made use of classic cell-type specific markers for astrocytes and microglia, based on human purified cell types shown in Figure 4A of Zhang et al. (2016).

### Summarizing cell type-specific marker expression in patch-seq and dissociated cell data

When directly comparing expression values from patch-seq data to dissociated cell data, we compared the Cadwell and Földy datasets to Tasic. These datasets each used Smart-seq-based methods for library preparation and mRNA counts were quantified using our in-house RNA-seq processing pipeline, with counts represented as transcripts per million (TPM + 1; plus 1 used to allow subsequent calculation of logarithms). Similarly, we compared the Fuzik dataset to Zeisel, as these both used single-cell tagged reverse transcription (C1-STRT) and quantified transcripts using unique molecule identifier counts (UMIs), which we normalized as UMI counts per million (UMI counts per million + 1).

We summarized the expression of multiple cell type specific markers specific to cell type *B (Markers*_*B*_), in a cell *c* of type *A* as:

$$\begin{array}{l} M_{c\text{\_}A,\;B}=\underset{m\in Markers_{B}}{\sum }log_{2}\left(c_{m}\right) \end{array}$$

Where *c*_*m*_ denotes the normalized expression of marker gene *m* in cell *c*.

We further used the dissociated-cell reference data to quantify how much marker expression of cell type *B*'s markers one would typically expect in cells of type *A* as:

$$\begin{array}{l} d_{A\text{\_}B}=median_{typeA}\left(M_{c\text{\_}A,\;B}\right) \end{array}$$

Reflecting the median marker expression of cell type *B*'s markers in dissociated cells of type *A*.

We defined the *contamination score* of markers of cell type *B* in a cell of type *A*. Specifically, given a patch-seq cell *c* of cell type *A* and markers of cell type B, we defined contamination score, *CS*_*A*_*B*_ as:

$$\begin{array}{l} CS_{A\text{\_}B}=\frac{M_{c\text{\_}A,\;B}-d_{A\text{\_}B}}{d_{B\text{\_}B}-d_{A\text{\_}B}} \end{array}$$

To elaborate on the contamination score, we first ask approximately how much marker expression of *B*'s markers one would expect in cells of type *A* using dissociated-cell data, and subtract this amount from *M*_*c*_*A*_, _*B*_. Since this value can be negative (for example, if cell *c* expresses none of *B*'s markers but *d*_*A*_*B*_ is positive), we set it to 0 in these cases (indicating that there is no detected contamination of cell type *B* in cell *c*). The denominator scales this value by the expected expression of *B*'s markers in cells of type *B*. Thus, *CS*_*A*_*B*_ reflects the expression of *B*'s markers in a cell of type *A*, relative to the expected expression of *B*'s markers in a cell of type *B*, based on dissociated-cell data. The contamination score can thus be intuitively interpreted as a ratio of the excess off-target marker expression, scaled between 0 and 1 (where 1 indicates the cell expresses the off-target cell type's markers at a level similar to the off-target cell type itself).

We defined the *contamination index* for cell *c* (of type *A*), reflecting off-target contamination across multiple broad cell types as:

$$\begin{array}{l} CI=\underset{t\in cell\;types,t\neq A}{\sum }CS_{t} \end{array}$$

As before when defining marker genes, when the patch-seq sample was a GABAergic cell type (e.g., Ndnf), we did not assess its contamination using markers of GABAergic types (e.g., Pvalb).

Lastly, to obtain a scalar *quality score* for a patch-seq cell c, we correlated each patch-seq sample's expression of “on” and “off” marker genes with the average expression profile of dissociated cells of the same type (Spearman correlation, shown in Supplementary Figure 2). For example, for a Ndnf patch-seq cell from Cadwell, we first calculated the average expression profile of Ndnf cells from Tasic across the set of all “on” and “off” marker genes (i.e., Ndnf markers, pyramidal cell markers, astrocyte markers, etc.), and then calculated the correlation between the patch-seq cell's marker expression to the mean dissociated cell expression profile. Since these correlations could potentially be negative, we set quality scores to a minimum of 0.1. A convenient feature of this quality score is that it yields low correlations for samples with relatively high off-target contamination as well as those where contamination is largely undetected but expression of endogenous “on” markers is also low (Supplementary Figure 2).

### Analysis of factors influencing the numbers of genes detected per cell

We analyzed how the following factors influenced the numbers of genes detected per cell:

- Library size, defined as the total count of sequenced reads per single-cell sample.
- Spike-in ratio, defined as the total number of reads mapping to the synthetic ERCC spike-in reference, divided by the library size.
- Unmapped ratio, defined as the total count of reads not mapping to either the transcriptomic or ERCC reference, divided by the library size minus ERCC count.
- Contamination index, reflecting off-target cell type contamination, as defined in the previous section.

For the Cadwell, Tasic, and ERCC-containing subsets of the Földy, and Bardy datasets, we fit a linear model (implemented using the “lm” function in R) for numbers of detected genes per each cell as follows:

$$\begin{array}{l} Detected\;gene\;count\;\sim \;library\;size+spike\;in\;ratio\\ \;+unmapped\;ratio+contam\;index \end{array}$$

where each term above was first scaled to z-scores, yielding standardized beta coefficients.

### Combined analysis of transcriptomic and electrophysiological features

We analyzed correlations between transcriptomic and electrophysiological features using an approach similar to our previous work (Tripathy et al., 2017). For each patch-seq dataset, we first filtered for genes whose average expression was >30th percentile relative to all genes in the dataset. We analyzed electrophysiological features overlapping with our previous analysis, specifically, input resistance (Rin), resting membrane potential (Vrest), action potential threshold (APthr), action potential amplitude (APamp), action potential half-width (APhw), membrane time constant (Tau), after-hyperpolarization amplitude (AHPamp), rheobase (Rheo), maximum firing rate (FRmax), and capacitance (Cm). We calculated Pearson correlations between the set of electrophysiology features and gene expression values, both without weighting cells by their overall quality scores (based on correlation of markers to dissociated cell samples), and after weighting cells using their quality scores.

We performed an analogous analysis for comparison of pooled-cell correlations based on the Allen Institute for Brain Sciences (AIBS) dataset, where we computationally merged different groups of cells characterized using dissociated cell scRNAseq [based on the Tasic dataset (Tasic et al., 2016)] with cells characterized using patch-clamp electrophysiology (Gouwens et al., 2018). We merged groups of cells based on the overlap of the same mouse transgenic lines and coarse cortical layers (i.e., upper vs. lower mouse visual cortex). For example, we merged 14 scRNAseq single-cells from the Sst-IRES-cre mouse line from visual cortex dissections specific to lower cortical layers with 89 patch-clamp cells from the same mouse line from cortical layers 4 through 6b. We averaged expression and electrophysiological values for cells from the same cell types (defined by cre-lines and cortical layers). Also, since cell types tended to be represented by differing numbers of cells (based on how these were sampled for scRNAseq or *in vitro* electrophysiology), in downstream analyses we weighted cell types based on the numbers of cells available characterized by electrophysiology, n_E_, and gene expression, n_G_, as:

$$\begin{array}{l} w=\sqrt{harmonic\;mean\left(n_{E},\;n_{G}\right)} \end{array}$$

where w denotes the weight for an individual cell type.

### Statistical information

We used the R weights toolbox (v0.85) to calculate weighted Pearson correlations and raw *p*-values. We used the Benjamini-Hochberg False Discovery Rate (FDR) to account for analysis of multiple correlations.

### Computer code and data availability

All computational code and associated data has been made accessible at https://github.com/PavlidisLab/patchSeqQC and code for the RNAseq pipeline is accessible at https://github.com/PavlidisLab/rnaseq-pipeline.

## Results

To quantitatively assess the influence of patch-seq specific technical confounds, we performed a re-analysis of five recently published patch-seq datasets. We focused our analyses on three datasets obtained from juvenile and adult mouse acute brain slices (Cadwell et al., 2015; Földy et al., 2016; Fuzik et al., 2016) and contrast these against two datasets obtained from human stem-cell derived neurons and astrocytes in culture (Bardy et al., 2016; Chen et al., 2016; Table 1). These datasets reflect, to our knowledge, all of the published patch-seq datasets as of October 2017. A major technical difference between the mouse and human patch-seq datasets re-analyzed here was that the former were collected from acute brain slices (with intact neuropil) whereas the latter were collected from cells that were sparsely cultured *in vitro*.

### Expression of off-target cell type marker genes in patch-seq samples from acute brain slices

**Figure 1:**
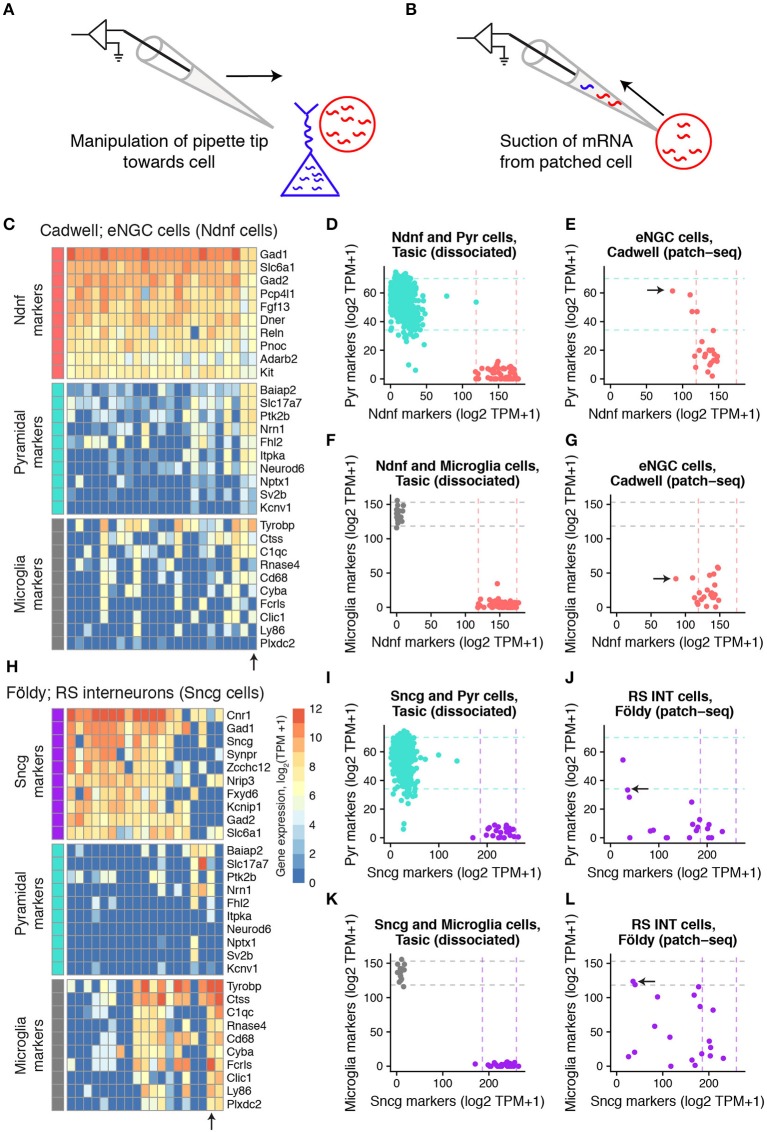
Expression of cell type-specific marker genes in mouse single-cell samples collected using patch-seq. **(A,B)** Schematic illustrating manipulation of patch-pipette toward cell of interest **(A)** and aspiration of cellular mRNA into the patch-pipette **(B)**. **(C)** Gene expression profiles for GABAergic elongated neurogliaform cells (eNGCs, similar to layer 1 Ndnf cellular subtype) for various cell type-specific markers characterized in Cadwell dataset. Each column reflects a single-cell sample. **(D)** Summed expression of cell type-specific marker genes for Pyramidal cell (y-axis) and Layer 1 Ndnf cell (x-axis) markers. Dots reflect Pyramidal (turquoise) and Ndnf (red) single cells collected in Tasic dataset, based on dissociated scRNAseq. Dashed lines reflect 95% intervals of marker expression for each cell type. **(E)** Same as **(D)**, but showing summed marker expression for eNGC cells shown in A based on patch-seq data. Arrow shows single-cell marked in **(C)**. **(F,G)** Same as **(D,E)**, but for microglial cell markers. **(H–L)** Same as **(C–G)**, but for hippocampal GABAergic regular spiking interneurons (RS INT cells, similar to Sncg cells from in Tasic) characterized in Földy dataset.

We first assessed if patch-seq based single-cell transcriptomes might have been contaminated by mRNA from other cells adjacent to the patched cell (Figures 1A,B), termed off-target cell-type contamination (Okaty et al., 2011). For example, is there paradoxical expression of genes specific to pyramidal cells in the scRNAseq profile of a recorded GABAergic interneuron? To address this question, we made use of the fact that the broad identities of the recorded cells can be ascertained from morphological and electrophysiological features without relying on the transcriptomic data (see Methods). Furthermore, we used multiple mouse forebrain scRNAseq datasets collected from dissociated cells to define lists of marker genes specific to various cortical and hippocampal cell types (Supplementary Table 4; Zeisel et al., 2015; Tasic et al., 2016; Mancarci et al., 2017).

We detected that some of the single cell samples from the three mouse datasets collected from acute brain slices expressed markers for multiple distinct cell types (Figure 1; Supplementary Figure 1). For example, some of the cortical layer 1 elongated neurogliaform cells (eNGCs) characterized in the Cadwell dataset appeared to also express multiple marker genes specific to pyramidal cells (Figure 1C), such as *Slc17a7*, the vesicular glutamatergic transporter VGLUT1. Similarly, many of the cells identified as hippocampal regular spiking GABAergic interneurons in the Földy dataset also expressed microglial and pyramidal cell markers (Figure 1H).

We sought to quantify the extent of off-target cell type contamination in the mouse patch-seq samples. We directly compared the patch-seq-based expression profiles to cellular dissociation-based transcriptomes from two recent surveys of mouse cortical diversity from Tasic et al. and Zeisel et al. (Zeisel et al., 2015; Tasic et al., 2016). We first quantified the levels of cell type-specific marker expression in the dissociated cells (see Methods, Figures 1D,F,I,K). After matching cell type identities across studies (shown in Supplementary Table 2), we found that compared to dissociated cells, patch-seq-based samples expressed markers for multiple cell types at considerably higher levels (Figures 1E,G,J,L).

**Figure 2:**
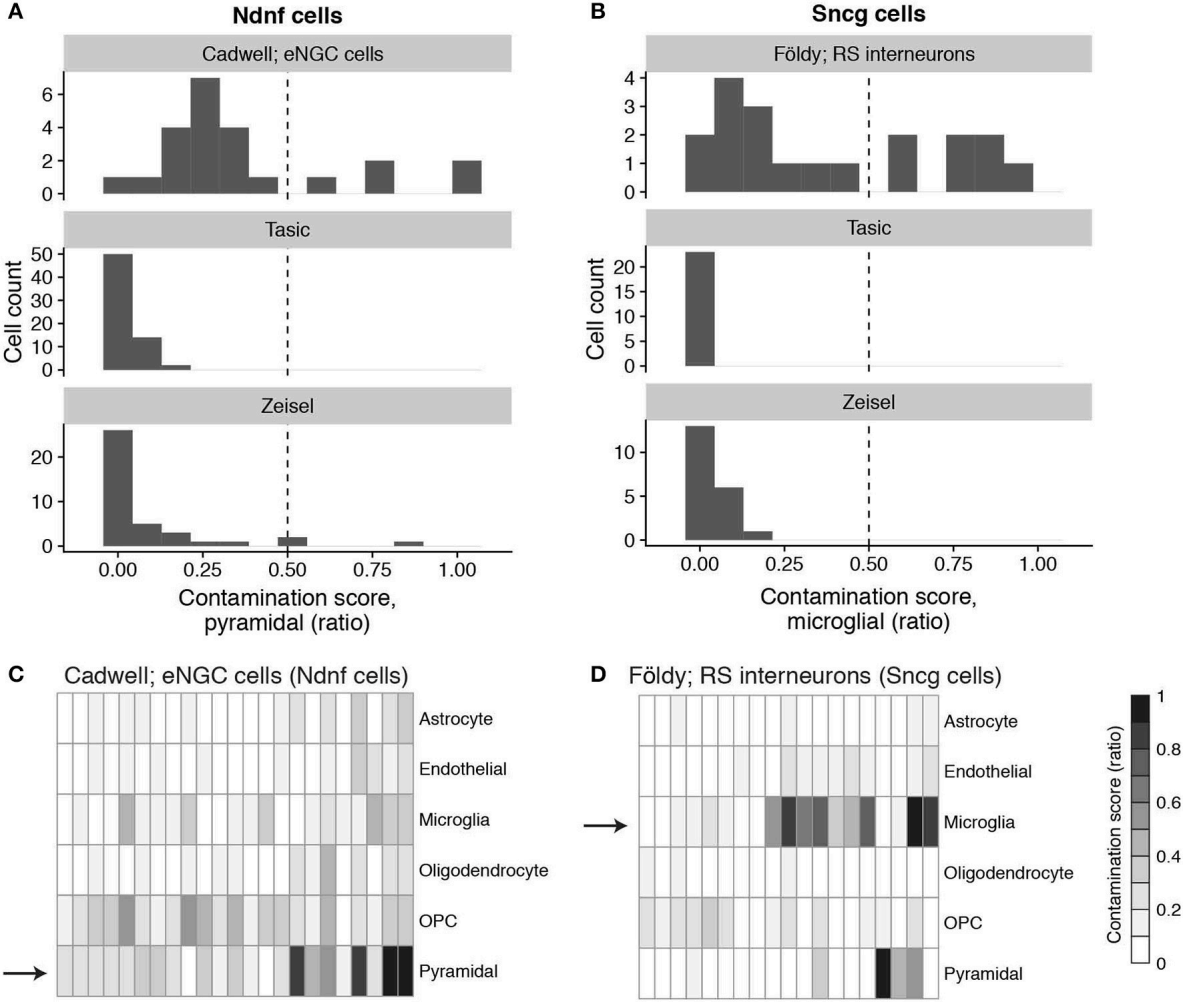
Cell type-specific contamination scores for patch-seq and dissociated cells. **(A)** Histogram of contamination scores of GABAergic elongated neurogliaform cells (eNGCs cells) from the Cadwell dataset (top) and analogous Ndnf cells from Tasic (middle) and Zeisel (bottom) dissociated-cell datasets. Contamination scores are specific to the expression of pyramidal cell type-specific marker genes. Dashed line at 0.5 indicates threshold used for calling single-cell contamination. **(B)** Same as **(A)**, but for hippocampal GABAergic regular spiking interneurons (RS INT cells) characterized in Földy dataset (top) and Sncg cells from Tasic and Zeisel datasets. **(C,D)** Contamination scores, indicated by heatmap color bar on right, across multiple classes of broad cell types for Cadwell eNGC cells **(C)** and Földy regular spiking interneurons **(D)**. Arrows denote same data shown in histograms in top panels of **(A,B)**.

To further quantify the extent of potential contamination, we defined a contamination score that evaluates single cell transcriptomes for off-target marker expression in comparison to dissociated cells (see Methods). In the top panel of Figure 2A we show the pyramidal marker contamination scores for eNGC interneurons from Cadwell. The contamination scores are scaled between 0 and 1, where 0 indicates no detected pyramidal contamination and 1 indicates the eNGC cell expresses pyramidal cell markers at levels similar to the median pyramidal cell in the Tasic dataset. We noticed that pyramidal marker contamination scores among the patch-seq cells from Cadwell were typically higher than those from the dissociated cell datasets (Figure 2A middle, bottom). We found that 5 out of 23 eNGC cells from Cadwell had pyramidal contamination scores greater than a threshold of 0.5 (see Discussion for further explanation for how such thresholds should be determined). Similarly, 7 out of 19 of the hippocampal regular spiking interneurons had microglial marker contamination scores >0.5 (Figure 2B). We calculated cell type-specific contamination scores for each broad cell type for every cell in the mouse patch-seq datasets (Figures 2C,D). In total, we found that 15 out of 57 cells in Cadwell (26%) and 16 out of 93 cells in Földy (17%) had single cells with contamination scores >0.5 among any of the broad cell types we tested.

Lastly, we defined a metric called the contamination index, that sums the contamination scores across multiple classes of broad cell types (see Methods). Importantly, patch-seq-based samples with greater contamination indices also expressed markers of their own cell type at lower levels (Supplementary Figure 2). We note that we saw less off-target cell type marker expression in the Fuzik dataset relative to the Cadwell and Földy datasets (Supplementary Figure 1), where only 1 out of 80 cells in Fuzik had a contamination score >0.5. This suggests either less contamination in the Fuzik dataset or that the lower gene detection rate in this dataset (Figure **4B**) may obscure our ability to use expression profiles to identify cellular contamination.

### Limited expression of off-target cell type marker genes among *in vitro* patch-seq samples from cultured cells

**Figure 3:**
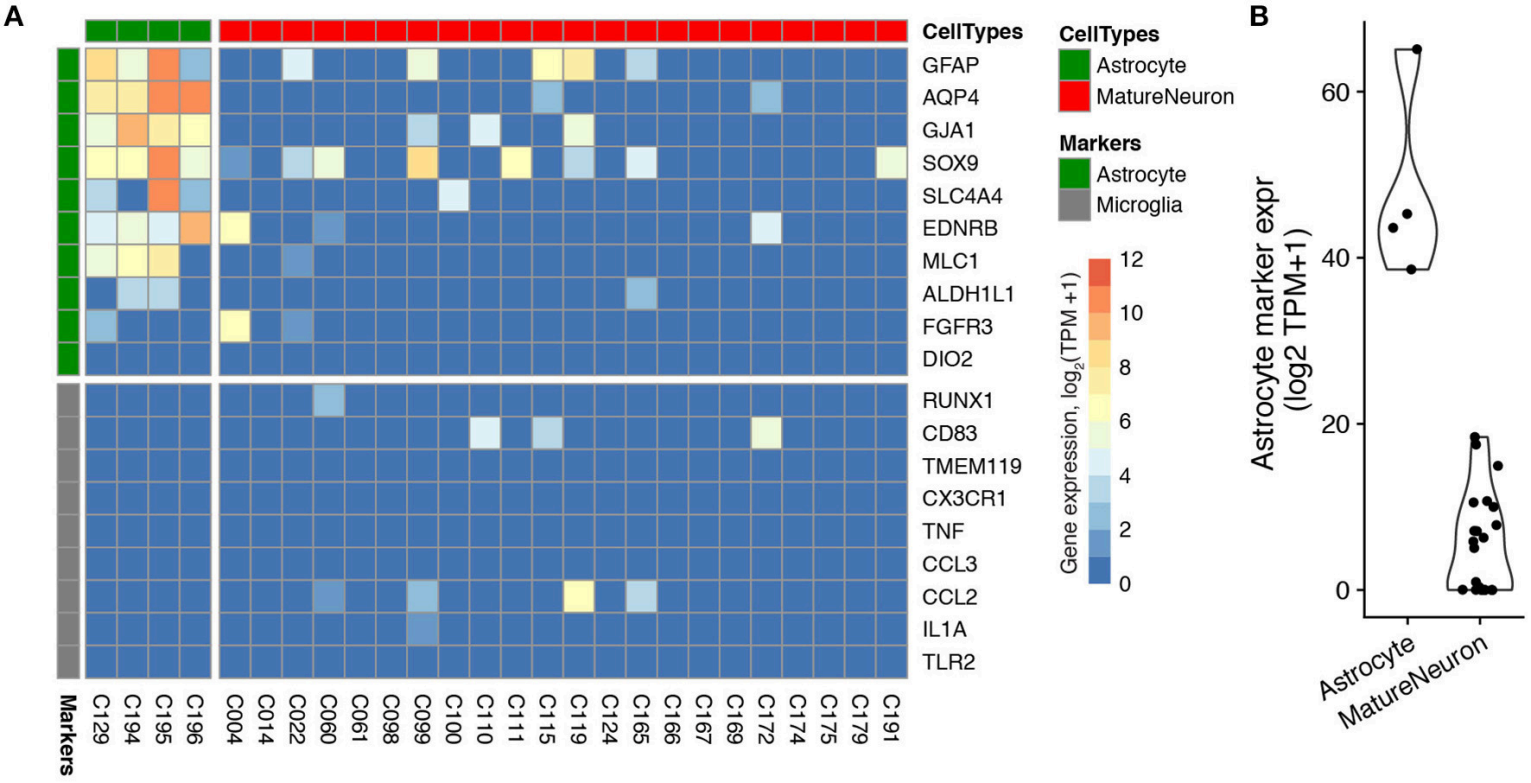
Expression of cell type-specific marker genes in patch-seq samples obtained from human astrocytes and neurons differentiated in culture from the Bardy dataset. **(A)** Gene expression profiles for differentiated astrocytes (green) and electrophysiologically-mature neurons (red) for astrocyte and microglial-specific (gray) marker genes. Each column reflects a single-cell sample. Two astrocyte cells were removed because they expressed fewer than three astrocyte markers. **(B)** Summed astrocyte marker expression for astrocyte and mature neuron single-cells, for the same cells shown in part **(A)**.

We next assessed the degree of off-target cell type contamination in the Bardy patch-seq dataset of human stem-cell derived neurons and astrocytes obtained from cultured cells (Bardy et al., 2016). Since the cells in this dataset were cultured relatively sparsely, allowing the processes of each cultured cell to be easily visualized and avoided during mRNA harvesting (personal communication with Cedric Bardy), we wondered if this dataset would show less off-target cell type marker expression compared to the three mouse acute brain slice datasets. Indeed, when assessing astrocyte marker expression in the population of electrophysiologically-mature neurons [with markers based on purified human cells (Zhang et al., 2016)], we found that every neuron showed much less expression of astrocyte markers relative to the mature astrocytes also profiled in this dataset (Figures 3A,B). In addition, both neurons and astrocytes showed almost no expression of microglia markers (Figure 3A), perhaps unsurprisingly, since microglial cells are not present in these cultures (Bardy et al., 2016). We also saw very little expression of astrocyte and microglial markers in electrophysiologically mature neurons from the Chen patch-seq dataset (Chen et al., 2016), another dataset of sparsely cultured neurons derived from human embryonic stem cells (Supplementary Figure 3). These examples provide suggestive evidence that the density of processes of adjacent cells might contribute to off-target mRNA contamination.

### Technical factors strongly influence the numbers of genes detected per cell

**Figure 4:**
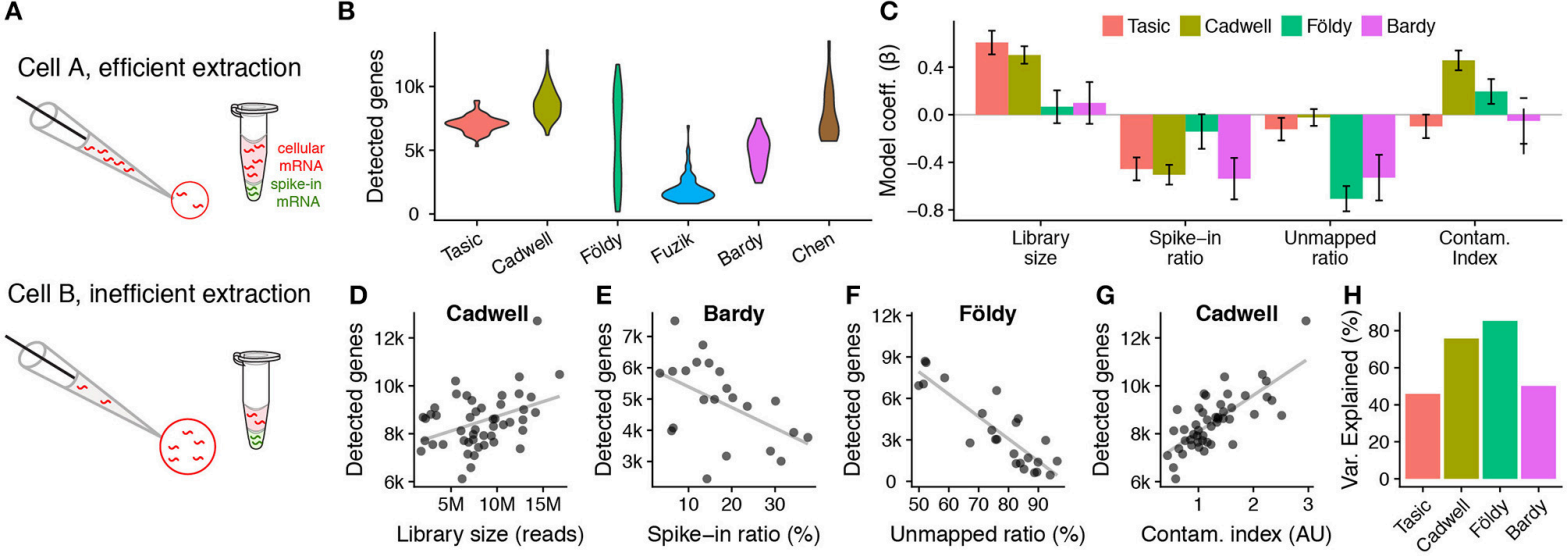
Patch-seq experimental confounds affect the numbers of genes detected per cell. **(A)** Schematic illustrating how spike-in mRNAs can be used to estimate how much mRNA was extracted per cell. **(B)** Violin plots showing numbers of protein-coding genes detected per cell across patch-seq datasets or the Ndnf subset of the Tasic dissociated-cell dataset. **(C)** Technical factors associated with numbers of genes detected per cell across datasets. Bars show standardized beta model coefficients with y-axis in units of standard deviations, allowing direct comparison of effects across factors and across datasets. Error bars indicate coefficient standard deviations. Positive (negative) model coefficients indicate technical factor is correlated with increased (decreased) detected gene counts per cell. Regression models calculated using only cells containing mRNA spike-ins. **(D–H)** Examples of univariate relationships between technical factors and detected gene count per cell (dots) across patch-seq datasets. Gray line shows best fit line. **(D)** Library size (count of sequenced reads per cell). **(E)** Spike-ins as a fraction of all sequenced reads per cell. Samples with lower cellular mRNA content (indicated by higher spike-in ratios) have lower gene counts. **(F)** Unmapped ratio, calculated as the ratio of exon-mapping reads to all sequenced reads (excluding spike-ins). **(G)** Cellular contamination index, quantified by summing normalized contamination scores across tested cell types (arbitrary units). **(H)** Overall percent variance explained by each dataset-specific statistical model shown in **(C)**.

Next, we wondered if there are identifiable technical factors that can help explain the large ranges in the numbers of genes detected per cell in each dataset, from 6,000–13,000 genes/cell in Cadwell to 800–7,000 genes/cell in Fuzik (Figure 4B). Because patch-seq mRNA collection requires the experimenter to manually aspirate cellular mRNA into the patch-pipette, we reasoned that mRNA harvesting would be difficult to consistently control from cell to cell, leading there to be different amounts of extracted mRNA per cell. To estimate how much cellular mRNA was extracted per cell, we made use of ERCC spike-ins (Tasic et al., 2017), which are synthetic control mRNAs that are added to single-cell samples prior to library preparation and sequencing (Figure 4A). Specifically, since the same amount of ERCC spike-in mRNAs are added to each sample, we can use the ratio of spike-in reads to the total count of sequenced reads to estimate the relative amount of extracted mRNA per cell (Lun et al., 2017; Vallejos et al., 2017). Here, every cell in the Cadwell and Tasic datasets and a subset of cells in the Földy and Bardy datasets contained ERCC spike-ins.

We used a multivariate regression approach to ask how various technical factors (library size, spike-in ratio, unmapped ratio, and contamination index, defined in Methods) contribute to the numbers of genes detected per cell (Figure 4C). We performed this analysis on the subset of cells containing ERCC spike-ins (we thus excluded the Fuzik and Chen datasets as these did not include spike-ins). As a comparison, we used the Ndnf cell subset of the Tasic dissociated-cell dataset, directly corresponding to the cell types sampled in the Cadwell dataset.

We found that library size (the number of sequenced reads per cell) was positively correlated with detected gene counts in the Tasic and Cadwell datasets (Figures 4C,D). Similarly, cells with a larger ratio of spike-in reads to total sequenced reads (i.e., with lower initial amounts of cellular mRNA; Figure 4A), had lower numbers of detected genes across all of the datasets (Figure 4E), pointing to the importance of mRNA extraction efficiency. In addition, we saw considerably greater ranges in the spike-in ratio in the patch-seq datasets relative to the Tasic dataset (Cadwell: 3–17%, Bardy: 3–37%, Tasic: 0.4–4%).

Next, we reasoned that though many mRNA transcripts might be extracted from a cell, not all of these would be sufficiently high quality to map to the genome or transcriptome reference [e.g., they might reflect degraded mRNAs (Cadwell et al., 2017a,b), other contaminants, etc.]. To account for this possibility, we calculated the ratio of unmapped to mapped reads, after excluding reads mapping to spike-ins. Cells with very large ratios of unmapped to mapped reads had fewer genes detected (Figure 4C). This technical factor was especially important in the Földy and Bardy datasets, with some cells in the Földy dataset having as many as 90% of reads being too low quality to map to the transcriptome (Figure 4F). Lastly, we further wondered if cells showing greater amounts of off-target cell type contamination would also have a greater number of detected genes. We found that cells with greater contamination indices from the Cadwell and Földy datasets (i.e., the acute slice-based patch-seq datasets) had more genes detected, consistent with previous reports (Figure 4G) (Ilicic et al., 2016; Vallejos et al., 2017). In total, these simple technical factors explain between 50 and 85% of the cell-to-cell variance in the detected gene counts per patch-seq dataset (Figure 4H).

### Accounting for technical factors improves the correspondence with electrophysiological features

**Figure 5:**
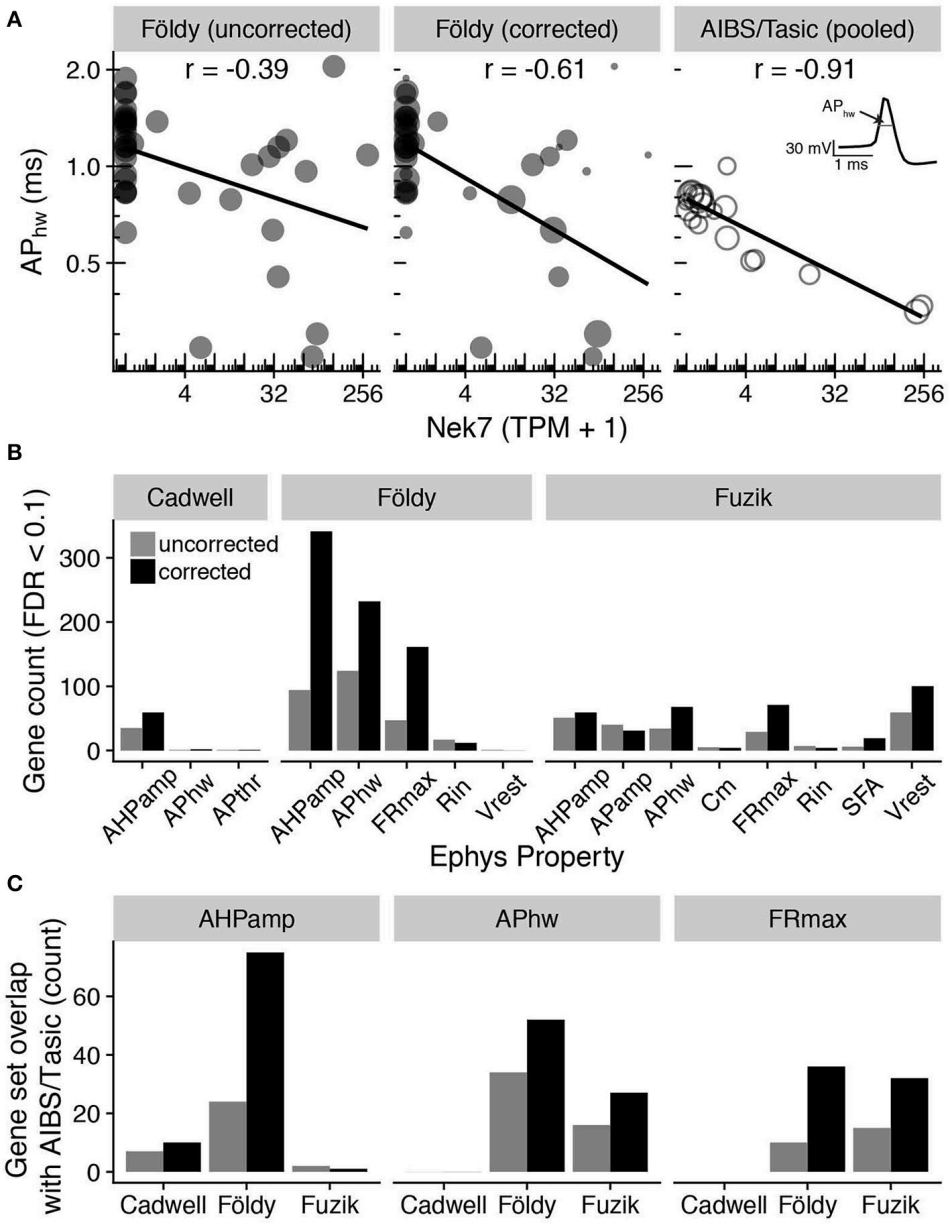
Adjusting for patch-seq experimental confounds improves the correspondence with electrophysiological measures. **(A)** Comparison of gene expression (Nek7; x-axis) with electrophysiological features (action potential half-width; APhw; y-axis). Left panel shows single-cell samples (circles) from the Földy dataset. Middle panel shows same data as left, but size of circles proportional to each sample's quality score, defined as the similarity of marker expression to dissociated cell-based reference data. Right panel shows cell type-level analysis based on pooled cell type data from Allen Institute cell types database (AIBS/Tasic), where scRNAseq and electrophysiology were performed on different cells from same type (Tripathy et al., 2017). Each open circle reflects one cell type and circle size is proportional to the number of cells representing each cell type. Inset illustrates calculation of action potential half-width (schematic). **(B)** Count of genes significantly correlated (FDR < 0.1) with various electrophysiological properties before (gray) and after (black) correcting for contamination by weighting cells by quality scores. See Methods for descriptions of electrophysiological features and acronyms. **(C)** Comparison of genes significantly correlated (FDR < 0.1) with electrophysiological features based on patch-seq data with analogous correlations based on AIBS/Tasic dataset, pooled to the level of cell types based on cre-lines. Bars indicate count of overlapping genes between patch-seq and AIBS/Tasic pooled-cell data without correcting for contamination and with correction. No maximum firing rate (FRmax) electrophysiological features were originally calculated for cells in the Cadwell dataset.

Lastly, we performed an integrated analysis of gene expression and electrophysiological features for the three mouse-based patch-seq datasets, reasoning that lower quality patch-seq samples would be less informative of relationships between cellular electrophysiology and gene expression (Tripathy et al., 2017). We first calculated a quality score for each patch-seq sampled cell, defined by the similarity of its marker expression profile to dissociated cells of its same type (see Methods; Supplementary Figure 2). We used this quality score as a weight in downstream correlation analyses, asking which genes had quantitative expression profiles that correlated with cell-to-cell variability in electrophysiological features. For example, the gene *Nek7* (NIMA-related expressed kinase 7), was more strongly correlated with the electrophysiological feature action potential half-width (APhw) in the Földy dataset after incorporating the quality score as a weight in the correlation analysis (Figure 5A left, middle; Pearson's r: −0.39, unweighted; −0.61, weighted). Performing an analogous correlation between *Nek7* expression and APhw using the Allen Institute for Brain Sciences (AIBS) Cell Types database / Tasic dataset at level of pooled cell types [see Methods (Tasic et al., 2016; Gouwens et al., 2018)], we also found a high degree of correlation between *Nek7* expression and APhw variability (Figure 5A right, r = −0.91). Intriguingly, the gene *Nek7* has recently been shown to be involved in the development of parvalbumin-positive interneurons (Hinojosa et al., 2018).

We repeated this correlation analysis across all pairs of genes and electrophysiological features, after accounting for multiple comparisons (using the Benjamini-Hochberg False Discovery Rate, FDR). We observed a modest improvement in the correspondence between gene expression and electrophysiology after incorporating the quality scores as a weight in the correlation analyses, as evidenced by an increase in the number of genes significantly correlated with electrophysiological features (FDR < 0.1, Figure 5B). In addition, we found more genes overlapping with those identified in our previous gene-electrophysiology correlation analysis based on pooled cell types using the AIBS/Tasic dataset (Tripathy et al., 2017; Figure 5C). While the biological implications of these gene-electrophysiological correlations require further investigation, this analysis suggests that controlling for these technical factors can help improve the interpretability of patch-seq data.

## Discussion

The patch-seq technique reflects a considerable leap in our ability to interrogate a neuron across multiple features of its function. However, our analyses of multiple patch-seq datasets identifies several technical issues that appeared to be shared across experiments. First, in the three mouse datasets collected from acute brain slices (Cadwell et al., 2015; Földy et al., 2016; Fuzik et al., 2016), we observed that many single cell samples appeared to strongly express marker genes from off-target cell types. We interpret this as mRNA contamination from cells adjacent to the recorded cell, though there are alternative explanations (see paragraph below). Second, we observed that mRNA extraction efficiency differs between sampled cells, leading to varying numbers of genes detected even among cells of the same broad type. These technical artifacts can be mitigated in part through *post-hoc* analyses, such as our attempt to define a quality score for weighting single-cells by the similarity of their marker gene expression to analogous dissociated cells of the same broad cell type.

To detect off-target cell type contamination, our main approach was to compare patch-seq based single-cell transcriptomes to dissociated-cell based reference scRNAseq data from similar cell types. We used these reference data to identify cell type-specific marker genes as well as to determine approximately how much off-target marker expression would be expected in each cell type. We note that there are obvious methodological differences between dissociated-cell scRNAseq and patch-seq (Cadwell et al., 2017a,b), such as the stress induced by dissociating cells (Wu et al., 2017) or that patch-seq might be more likely to sample transcripts from distal cellular processes. Thus, we cannot conclusively rule out that some of the off-target cell type marker expression might reflect a true biological signal, as opposed to mRNA contamination from adjacent cells. However, we note that the use of marker genes to identify suspected off-target contamination is a routine quality control step in cell type-specific gene expression analyses (Okaty et al., 2011; Mancarci et al., 2017; Pfeffer and Beltramo, 2017), including recent methods for identifying suspected “doublets” or multi-cell contamination in droplet-based scRNAseq (Zeisel et al., 2018).

We speculate that the sources of off-target contamination are the processes of cells adjacent to the patch-pipette. For example, while there are relatively few cell bodies in layer 1 of the neocortex, there are processes of other cell types like pyramidal cells, and it is well established that these processes contain mRNA transcripts (Glock et al., 2017). In addition, we noticed that we routinely observed expression of microglial markers in the acute slice-based mouse patch-seq samples. This is interesting because the presence of even 1 mM ATP in the patch-pipette is sufficient to induce rapid chemotaxis of microglial processes toward the pipette (Madry et al., 2018). Patch-clamp intracellular solutions usually use 2 or 4 mM ATP (Tebaykin et al., 2017), including those of the patch-seq datasets here (Cadwell et al., 2015; Bardy et al., 2016; Földy et al., 2016; Fuzik et al., 2016). At present, it is unclear whether this suspected off-target contamination might occur while the pipette is actively manipulated under positive pressure toward the recorded cell. Alternatively, such contamination might take place following mRNA extraction during the retraction of the pipette from the neuropil and recording chamber. Contamination might also occur during library preparation (Pfeffer and Beltramo, 2017). Assuming that the neuropil is the major source of off-target contamination, this suggests that there may be advantages to removing the neuropil prior to performing patch-seq. For example, patch-seq can be performed on sparsely cultured cells (Bardy et al., 2016; Chen et al., 2016; van den Hurk et al., 2018). Similarly, given that there are well established protocols for performing electrophysiological characterization on acutely dissociated cells (Swensen and Bean, 2005) that allow for subsequent cytoplasmic mRNA harvesting (Kodama et al., 2012), patch-seq could in principle be performed following acute cellular dissociation.

Our analyses identified several technical factors that influence the numbers of genes detected per cell. First, to obtain a sufficient number of detected genes, it is essential to extract a large amount of mRNA from the targeted cell. For example, extracting the nucleus can help increase the detected gene count (Cadwell et al., 2017b). However, this itself is not sufficient, as other factors, such as mRNA degradation can lead the extracted transcripts being too low quality to map to the genomic reference (Cadwell et al., 2017a,b; van den Hurk et al., 2018). Second, given sufficient extraction of non-degraded transcripts, because of the extremely high sensitivity of modern ultra-low mRNA capture kits (Poulin et al., 2016; Tasic et al., 2017), any off-target cell-type contamination will tend to inflate the numbers of genes detected per cell. This suggests that the detected gene count, often used as a proxy for the quality of scRNAseq data, should not be the only quality control metric for single-cell transcriptomes sampled using patch-seq.

The effect of these technical confounds on downstream analyses of patch-seq data is likely context specific. For example, the presence of a small degree of off-target contamination is likely to be of little consequence if the patch-seq data is used for catalog-matching (Tasic et al., 2017), to help connect cellular classifications based on different methodologies, such as transcriptomically-defined cell clusters with electrophysiological clusters (Fuzik et al., 2016; Muñoz-Manchado et al., 2018). However, accurately quantifying single-cell transcriptomes is likely to be much more important when using these data to investigate how transcriptomic heterogeneity gives rise to subtle cell to cell variability in physiological features (Schulz et al., 2006; Cadwell et al., 2015; Tripathy et al., 2017).

Our analyses point to quality control steps that can improve the yield of high-quality patch-seq samples. An advantage of patch-seq over traditional dissociated-cell based scRNA-seq is that a cell's electrophysiological and morphological features are often sufficient to determine its broad cell type (Cadwell et al., 2015; Földy et al., 2016; Fuzik et al., 2016). Furthermore, knowing a cell's broad type can help quality control its sampled transcriptome. Namely, the cell should express marker genes of its own type, including highly expressed and lowly expressed markers (such as some transcription factors and long non-coding RNAs) but should not express marker genes specific to other cell types. We argue that *post-hoc* assessment of cell type-specific marker expression should be routinely reported in future patch-seq studies as a quality control metric. In addition, marker expression could also be assessed as an intermediate quality control step. For example, in a recent patch-seq protocol (van den Hurk et al., 2018), the authors suggest using qRT-PCR (in step 89) to ensure that collected cells have high expression of house-keeping genes (e.g., ACTB, GAPDH) prior to subsequent library preparation. We hypothesize that qRT-PCR could also be employed at this step to profile the expression of a small number of cell type-specific markers, allowing cells that have been potentially contaminated by off-target mRNA to be detected and removed prior to subsequent steps, including costly deep RNA-sequencing.

To summarize, though patch-seq provides a powerful method for multi-modal neuronal characterization (Cadwell et al., 2015; Bardy et al., 2016; Chen et al., 2016; Földy et al., 2016; Fuzik et al., 2016), it is susceptible to a number of methodology-specific technical artifacts, such as an increased likelihood of mRNA contamination from adjacent cells. These artifacts strongly bias traditional scRNAseq quality metrics, such as the numbers of genes detected per cell. By leveraging high-quality reference atlases of single-cell transcriptomic diversity (Zeisel et al., 2015; Tasic et al., 2016), we argue that inspection of cell type-specific marker expression should be an essential patch-seq quality control step prior to downstream analyses.

## Author contributions

ST and PP conceived the project. ST implemented the methodology and generated the results with assistance from LT, BM, CB, and MB. All authors contributed to interpreting the results. ST and PP wrote the paper with assistance from all authors.

### Conflict of interest statement

The authors declare that the research was conducted in the absence of any commercial or financial relationships that could be construed as a potential conflict of interest. The reviewer LT and handling editor declared their shared affiliation at time of review.

## Supplementary material

Supplementary Figure 1 Expression of cell type-specific marker genes in patch-seq samples from Fuzik. **(A)** Gene expression profiles for sampled pyramidal cells for various cell type-specific markers. **(B)** Summed expression of cell type-specific marker genes for Pyramidal cell (x-axis) and Astrocyte (y-axis) markers. Dots reflect cortical Pyramidal cell (turquoise) and Astrocyte (green) single cells collected in the Zeisel dataset, based on dissociated scRNAseq. Dashed lines reflect 95% intervals of marker expression for each cell type. **(C)** Same as **(B)**, but showing summed marker expression for Pyramidal cells shown in **(A)** based on patch-seq data. Arrow denotes the same single-cell highlighted in **(A)**. **(D,E)** Same as **(B,C)**, but showing comparison of microglial marker expression. **(F)** Contamination scores across multiple classes of broad cell types for same cells shown in **(A)**. Oligodendrocyte precursor cells are not available in **(F)** because this cell type was not explicitly annotated in the Zeisel dataset.

Supplementary Figure 2 Relationship between inferred contamination and endogenous marker expression. **(A)** Summed expression of endogenous “on”-cell type cellular markers (x-axis) vs. normalized contamination indices (y-axis, summing across normalized contamination values across broad cell types) for individual Ndnf cells from the Cadwell dataset (dots). **(B,C)** Examples of “on”- and “off”-cell type marker expression for two single-cell patch-seq samples indicated in **(A)**. X-axis shows expression of marker genes (dots) in an individual patch-seq sampled cell and y-axis shows the average expression of the same markers in Ndnf-type dissociated cells from Tasic. Solid line is unity line, dashed line shows best linear fit, and r_s_ denotes Spearman correlation between patch-seq and mean dissociated cell marker expression. Cell Ndnf.1 [shown in **(B)**] illustrates a patch-seq sample with high expression of “on”-type endogenous markers and relatively little “off”-cell type marker expression whereas cell Ndnf.2 [shown in **(C)**] expresses endogenous markers less strongly (relative to dissociated cells of same type) and higher levels “off”-cell type marker expression. **(D–F)** Same as **(A–C)**, but for hippocampal GABAergic regular spiking interneurons (i.e., Sncg cells) characterized in Földy dataset.

Supplementary Figure 3 Expression of cell type-specific marker genes in patch-seq samples obtained from human neurons differentiated in culture from the Chen dataset. Gene expression profiles for electrophysiologically-mature neurons (red) for astrocyte (green) and microglial-specific (gray) marker genes. Each column reflects a single-cell sample. Gene expression values are quantified as fragments per kilobase per million (FPKM).

Supplementary Table 1 Description of dissociated-cell scRNAseq datasets and patch-clamp electrophysiological datasets used. For RNA amplification, the Tasic scRNAseq dataset employed SMARTer (i.e., Smart-seq based, consistent with the Cadwell, Foldy, and Bardy datasets) whereas the Zeisel dataset employed C1-STRT (consistent with the Fuzik dataset).

Supplementary Table 2 Matching of patch-seq cell types to dissociated cell reference atlases.

Supplementary Table 3 Mapping of broad cell types between Tasic and Zeisel dissociated cell reference datasets. ^*^Denotes oligodendrocyte precursor cell type not being explicitly labelled in Zeisel.

Supplementary Table 4 List of cell type-specific markers based on re-analysis of published dissociated cell-based scRNAseq experiments from mouse brain.
