# Supplementary figures and images for: Assessing Transcriptome Quality in Patch-Seq Datasets
Source: Front Mol Neurosci. 2018 Oct 8;11:363. doi: 10.3389/fnmol.2018.00363 (PMC6187980; doi:10.3389/fnmol.2018.00363)

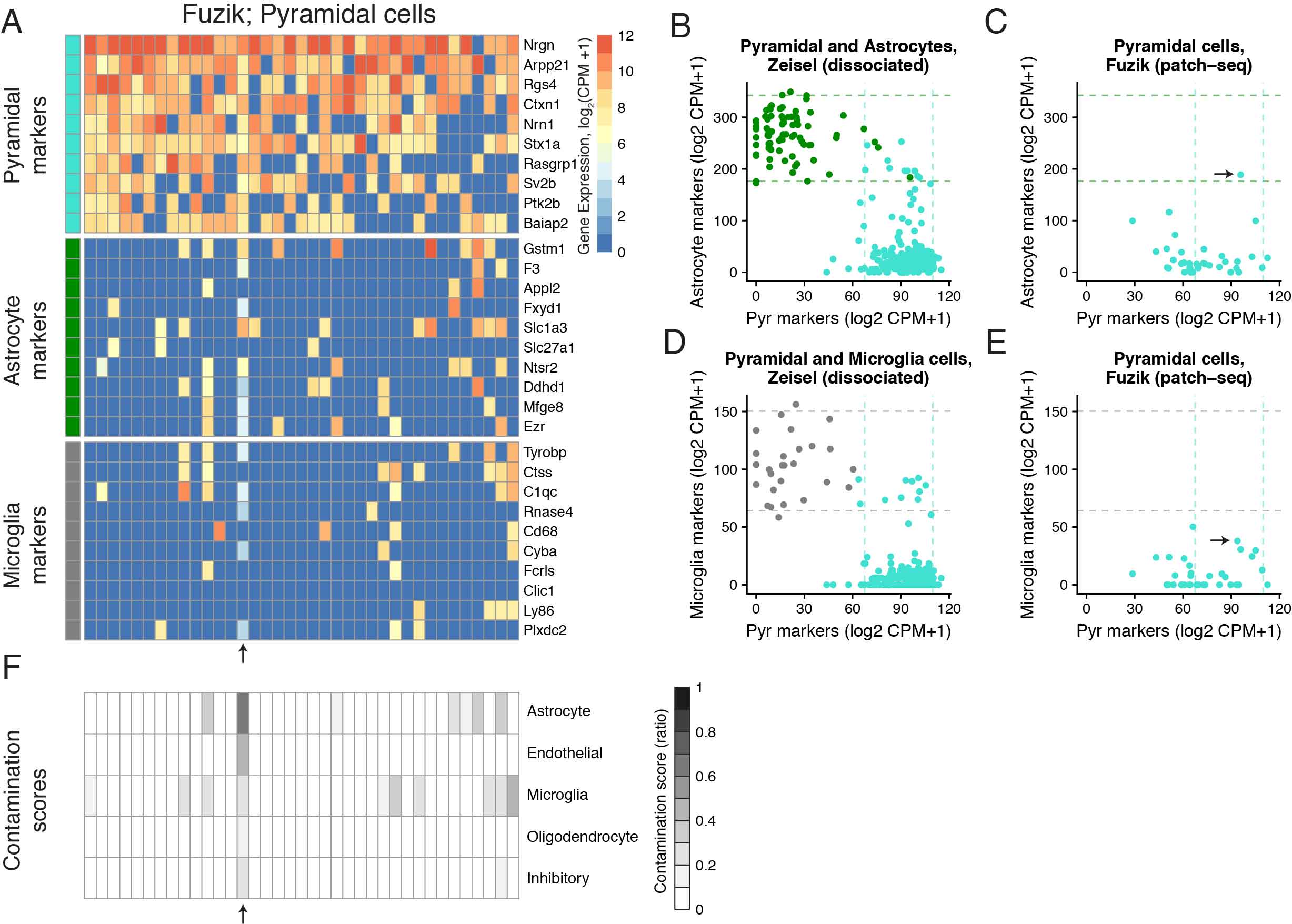

Supplement: Supplementary Figure 1 — Expression of cell type-specific marker genes in patch-seq samples from Fuzik. (A) Gene expression profiles for sampled pyramidal cells for various cell type-specific markers. (B) Summed expression of cell type-specific marker genes for Pyramidal cell (x-axis) and Astrocyte (y-axis) markers. Dots reflect cortical Pyramidal cell (turquoise) and Astrocyte (green) single cells collected in the Zeisel dataset, based on dissociated scRNAseq. Dashed lines reflect 95% intervals of marker expression for each cell type. (C) Same as (B), but showing summed marker expression for Pyramidal cells shown in (A) based on patch-seq data. Arrow denotes the same single-cell highlighted in (A). (D,E) Same as (B,C), but showing comparison of microglial marker expression. (F) Contamination scores across multiple classes of broad cell types for same cells shown in (A). Oligodendrocyte precursor cells are not available in (F) because this cell type was not explicitly annotated in the Zeisel dataset. [file Image_1.JPEG]

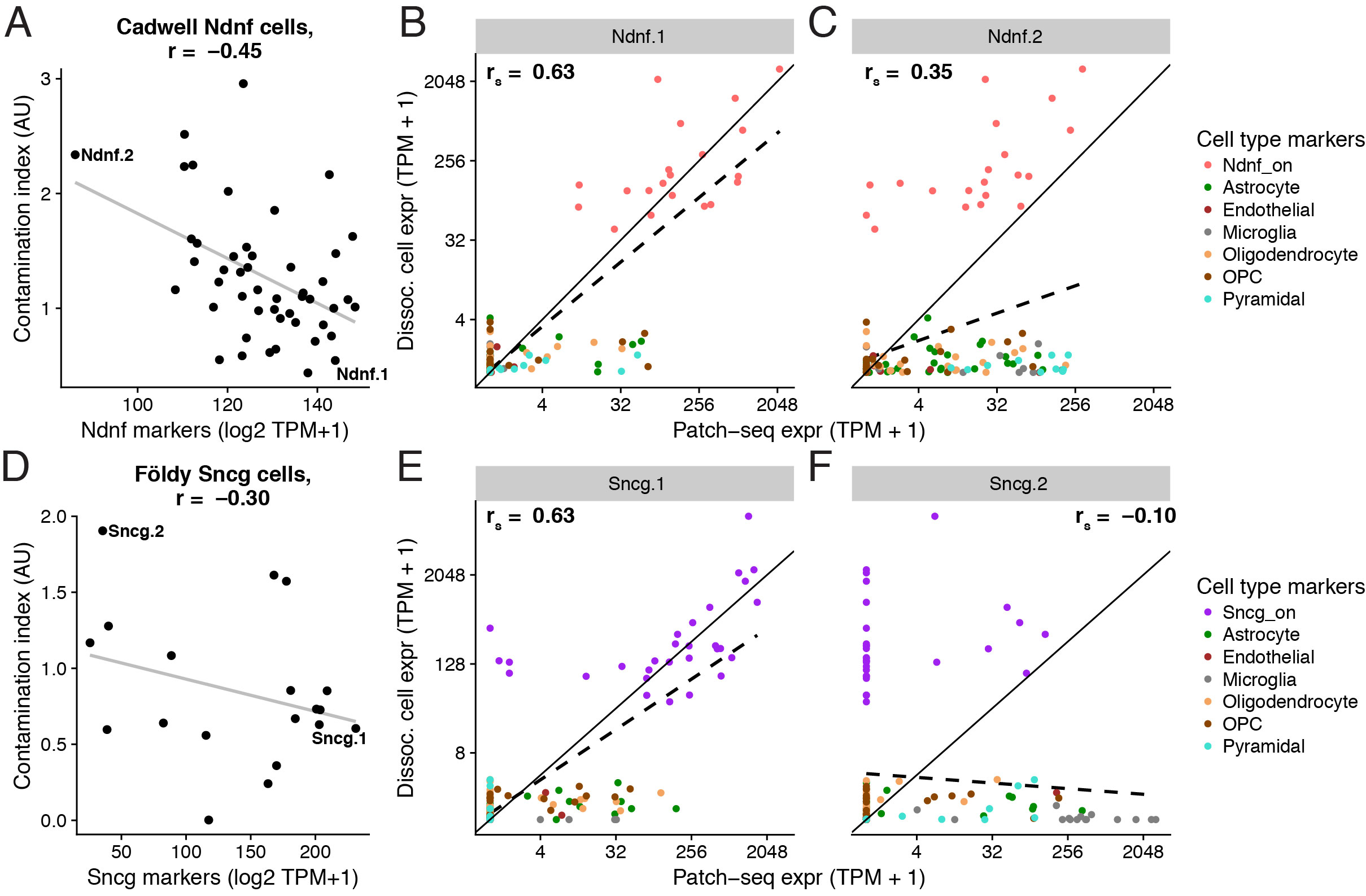

Supplement: Supplementary Figure 2 — Relationship between inferred contamination and endogenous marker expression. (A) Summed expression of endogenous “on”-cell type cellular markers (x-axis) vs. normalized contamination indices (y-axis, summing across normalized contamination values across broad cell types) for individual Ndnf cells from the Cadwell dataset (dots). (B,C) Examples of “on”- and “off”-cell type marker expression for two single-cell patch-seq samples indicated in (A). X-axis shows expression of marker genes (dots) in an individual patch-seq sampled cell and y-axis shows the average expression of the same markers in Ndnf-type dissociated cells from Tasic. Solid line is unity line, dashed line shows best linear fit, and rs denotes Spearman correlation between patch-seq and mean dissociated cell marker expression. Cell Ndnf.1 [shown in (B)] illustrates a patch-seq sample with high expression of “on”-type endogenous markers and relatively little “off”-cell type marker expression whereas cell Ndnf.2 [shown in (C)] expresses endogenous markers less strongly (relative to dissociated cells of same type) and higher levels “off”-cell type marker expression. (D–F) Same as (A–C), but for hippocampal GABAergic regular spiking interneurons (i.e., Sncg cells) characterized in Földy dataset. [file Image_2.JPEG]

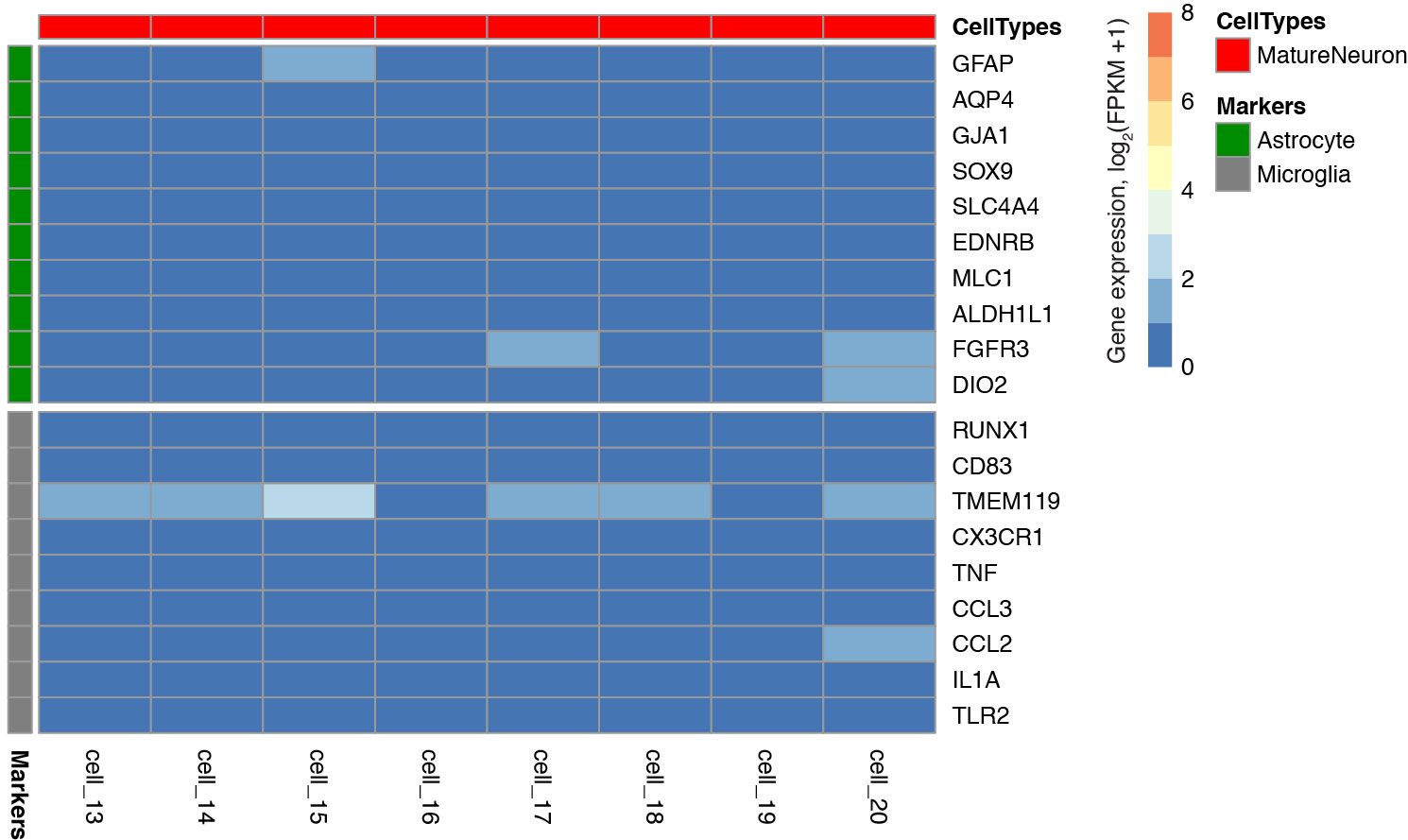

Supplement: Supplementary Figure 3 — Expression of cell type-specific marker genes in patch-seq samples obtained from human neurons differentiated in culture from the Chen dataset. Gene expression profiles for electrophysiologically-mature neurons (red) for astrocyte (green) and microglial-specific (gray) marker genes. Each column reflects a single-cell sample. Gene expression values are quantified as fragments per kilobase per million (FPKM). [file Image_3.JPEG]
