# Supplementary material for: Assessing Transcriptome Quality in Patch-Seq Datasets
Source: Front Mol Neurosci. 2018 Oct 8;11:363. doi: 10.3389/fnmol.2018.00363 (PMC6187980; doi:10.3389/fnmol.2018.00363)
Supplement: Supplementary Table 1 — Description of dissociated-cell scRNAseq datasets and patch-clamp electrophysiological datasets used. For RNA amplification, the Tasic scRNAseq dataset employed SMARTer (i.e., Smart-seq based, consistent with the Cadwell, Foldy, and Bardy datasets) whereas the Zeisel dataset employed C1-STRT (consistent with the Fuzik dataset). [file Data_Sheet_2.docx]

**Supplementary Tables**

| **Dataset** | **Experiment type** | **Preparation** | **Description** | **Accession** | **Number of cells** |
| --- | --- | --- | --- | --- | --- |
| Tasic (Tasic et al., 2016) | Dissociated cell scRNAseq | Dissociated cells | Visual cortex neurons and glia | GSE71585 | 1366 |
| Zeisel (Zeisel et al., 2015) | Dissociated cell  scRNAseq | Dissociated cells | Somatosensory cortex and hippocampus neurons and glia | GSE60361 | 3005 |
| Allen Institute Cell Types (Gouwens et al., 2018) | Patch-clamp electrophysiology | Acute mouse slices | Visual cortex neurons | celltypes.brain-map.org | 952 |

Supplementary Table 1: Description of dissociated-cell scRNAseq datasets and patch-clamp electrophysiological datasets used. For RNA amplification, the Tasic scRNAseq dataset employed SMARTer (i.e., Smart-seq based, consistent with the Cadwell, Foldy, and Bardy datasets) whereas the Zeisel dataset employed C1-STRT (consistent with the Fuzik dataset).

| **Patch-seq dataset** | **Cell type**  **(patch-seq)** | **Matched cell type (dissociated cell; Tasic)** | **Matched cell type (dissociated cell; Zeisel)** |
| --- | --- | --- | --- |
| Cadwell | Cortex Layer 1 elongated neuragliaform cell  (eNGC) | Ndnf cluster (Ndnf Car4, Ndnf Cxcl14) | Int12, Int15 |
| Cadwell | Cortex Layer 1 single bouquet cells (SBC) | Ndnf cluster (Ndnf Car4, Ndnf Cxcl14) | Int12, Int15 |
| Földy | Hippocampus regular-spiking (RS) interneurons | Sncg | Int 5 |
| Földy | Hippocampus CA1 and Subiculum Pyramidal cells | Pyramidal cluster | Pyramidal cluster (excluding CA1PyrInt) |
| Földy | Hippocampus fast-spiking (FS) interneurons | Pvalb cluster (Pvalb Gpx3, Pvalb Wt1, Pvalb Tacr3, Pvalb Tpbg, Pvalb Cpne5, Pvalb Rspo2, Pvalb Obox3) | Int 3 |
| Fuzik | Cortex Layer 1 and 2 interneurons | Ndnf cluster (Ndnf Car4, Ndnf Cxcl14) | Int12, Int15 |
| Fuzik | Cortex Pyramidal cells | Pyramidal cluster | Pyramidal cluster (excluding CA1PyrInt) |

Supplementary Table 2: Matching of patch-seq cell types to dissociated cell reference atlases.

| **Broad cell type** | **Tasic subtypes** | **Zeisel subtypes** |
| --- | --- | --- |
| Astrocyte | Astro Gja1 | Astro2, Astro1 |
| Endothelial | Endo Myl9, Endo Tbc1d4 | Vsmc |
| Inhibitory | Vip Chat, Vip Parm1, Vip Mybpc1, Vip Gpc3, Pvalb Gpx3, Ndnf Cxcl14, Vip Sncg, Ndnf Car4, Sst Myh8, Sst Th, Sst Chodl, Sst Tacstd2, Sst Cdk6, Pvalb Wt1, Sncg, Sst Cbln4, Pvalb Tacr3, Igtp, Smad3, Pvalb Tpbg, Pvalb Cpne5, Pvalb Rspo2, Pvalb Obox3 | Int10, Int6, Int9, Int2, Int4, Int1, Int3, Int13, Int16, Int14, Int11, Int5, Int7, Int8, Int12, Int15 |
| Microglia | Micro Ctss | Mgl1, Mgl2 |
| Oligodendrocyte | Oligo Opalin, Oligo 96_Rik | Oligo1, Oligo3, Oligo4, Oligo2, Oligo6, Oligo5 |
| OPC | OPC Pdgfra | * |
| Pyramidal | L2/3 Ptgs2, L2 Ngb, L4 Ctxn3, L4 Scnn1a, L5a Batf3, L5a Pde1c, L6a Mgp, L6b Serpinb11, L6b Rgs12, L5a Hsd11b1, L4 Arf5, L5a Tcerg1l, L6a Sla, L6a Syt17, L6a Car12, L5b Cdh13, L5 Ucma, L5b Tph2, L5 Chrna6 | S1PyrL4, ClauPyr, S1PyrL5, S1PyrL23, S1PyrDL, S1PyrL5a, SubPyr, CA1Pyr1, S1PyrL6b, S1PyrL6, CA1Pyr2, CA2Pyr2 |

Supplementary Table 3. Mapping of broad cell types between Tasic and Zeisel dissociated cell reference datasets. * denotes oligodendrocyte precursor cell type not being explicitly labelled in Zeisel.

|  | **Cell Type** | **Gene name** | **Ensembl Gene ID** | **Entrez gene ID** |
| --- | --- | --- | --- | --- |
| 1 | Astrocyte | Gstm1 | ENSMUSG00000058135 | 14862 |
| 2 | Astrocyte | F3 | ENSMUSG00000028128 | 14066 |
| 3 | Astrocyte | Appl2 | ENSMUSG00000020263 | 216190 |
| 4 | Astrocyte | Fxyd1 | ENSMUSG00000036570 | 56188 |
| 5 | Astrocyte | Slc1a3 | ENSMUSG00000005360 | 20512 |
| 6 | Astrocyte | Slc27a1 | ENSMUSG00000031808 | 26457 |
| 7 | Astrocyte | Ntsr2 | ENSMUSG00000020591 | 18217 |
| 8 | Astrocyte | Ddhd1 | ENSMUSG00000037697 | 114874 |
| 9 | Astrocyte | Mfge8 | ENSMUSG00000030605 | 17304 |
| 10 | Astrocyte | Ezr | ENSMUSG00000052397 | 22350 |
| 11 | Astrocyte | Naaa | ENSMUSG00000029413 | 67111 |
| 12 | Astrocyte | Acsbg1 | ENSMUSG00000032281 | 94180 |
| 13 | Astrocyte | S1pr1 | ENSMUSG00000045092 | 13609 |
| 14 | Astrocyte | Sox9 | ENSMUSG00000000567 | 20682 |
| 15 | Astrocyte | Sdc4 | ENSMUSG00000017009 | 20971 |
| 16 | Astrocyte | Aldh1l1 | ENSMUSG00000030088 | 107747 |
| 17 | Astrocyte | Lcat | ENSMUSG00000035237 | 16816 |
| 18 | Astrocyte | Slc38a3 | ENSMUSG00000010064 | 76257 |
| 19 | Astrocyte | Cldn10 | ENSMUSG00000022132 | 58187 |
| 20 | Astrocyte | Id4 | ENSMUSG00000021379 | 15904 |
| 21 | Astrocyte | Mlc1 | ENSMUSG00000035805 | 170790 |
| 22 | Astrocyte | Fgfr3 | ENSMUSG00000054252 | 14184 |
| 23 | Astrocyte | Slco1c1 | ENSMUSG00000030235 | 58807 |
| 24 | Astrocyte | Paqr8 | ENSMUSG00000025931 | 74229 |
| 25 | Astrocyte | Cbs | ENSMUSG00000024039 | 12411 |
| 26 | Astrocyte | Prodh | ENSMUSG00000003526 | 19125 |
| 27 | Astrocyte | Phkg1 | ENSMUSG00000025537 | 18682 |
| 28 | Astrocyte | Adhfe1 | ENSMUSG00000025911 | 76187 |
| 29 | Astrocyte | Slc15a2 | ENSMUSG00000022899 | 57738 |
| 30 | Astrocyte | Tril | ENSMUSG00000043496 | 66873 |
| 31 | Astrocyte | Dio2 | ENSMUSG00000007682 | 13371 |
| 32 | Astrocyte | Arhgef26 | ENSMUSG00000036885 | 622434 |
| 33 | Astrocyte | Cyp2d22 | ENSMUSG00000061740 | 56448 |
| 34 | Astrocyte | Aqp4 | ENSMUSG00000024411 | 11829 |
| 35 | Astrocyte | Smpdl3a | ENSMUSG00000019872 | 57319 |
| 36 | Astrocyte | Cyp2j9 | ENSMUSG00000015224 | 74519 |
| 37 | Astrocyte | Vcam1 | ENSMUSG00000027962 | 22329 |
| 38 | Astrocyte | Gabrg1 | ENSMUSG00000001260 | 14405 |
| 39 | Astrocyte | Slc39a12 | ENSMUSG00000036949 | 277468 |
| 40 | Endothelial | Crip1 | ENSMUSG00000006360 | 12925 |
| 41 | Endothelial | Tpm4 | ENSMUSG00000031799 | 326618 |
| 42 | Endothelial | Arhgap29 | ENSMUSG00000039831 | 214137 |
| 43 | Endothelial | Igfbp7 | ENSMUSG00000036256 | 29817 |
| 44 | Endothelial | S100a11 | ENSMUSG00000027907 | 20195 |
| 45 | Endothelial | Tm4sf1 | ENSMUSG00000027800 | 17112 |
| 46 | Endothelial | Col4a1 | ENSMUSG00000031502 | 12826 |
| 47 | Endothelial | Esam | ENSMUSG00000001946 | 69524 |
| 48 | Endothelial | Ahnak | ENSMUSG00000069833 | 66395 |
| 49 | Microglia | Tyrobp | ENSMUSG00000030579 | 22177 |
| 50 | Microglia | Ctss | ENSMUSG00000038642 | 13040 |
| 51 | Microglia | C1qc | ENSMUSG00000036896 | 12262 |
| 52 | Microglia | Rnase4 | ENSMUSG00000021876 | 58809 |
| 53 | Microglia | Cd68 | ENSMUSG00000018774 | 12514 |
| 54 | Microglia | Cyba | ENSMUSG00000006519 | 13057 |
| 55 | Microglia | Fcrls | ENSMUSG00000015852 | 80891 |
| 56 | Microglia | Clic1 | ENSMUSG00000007041 | 114584 |
| 57 | Microglia | Ly86 | ENSMUSG00000021423 | 17084 |
| 58 | Microglia | Plxdc2 | ENSMUSG00000026748 | 67448 |
| 59 | Microglia | Entpd1 | ENSMUSG00000048120 | 12495 |
| 60 | Microglia | Lyn | ENSMUSG00000042228 | 17096 |
| 61 | Microglia | C3ar1 | ENSMUSG00000040552 | 12267 |
| 62 | Microglia | Cfh | ENSMUSG00000026365 | 12628 |
| 63 | Microglia | Hk2 | ENSMUSG00000000628 | 15277 |
| 64 | Microglia | Zfhx3 | ENSMUSG00000038872 | 11906 |
| 65 | Oligodendrocyte | Desi1 | ENSMUSG00000022472 | 28075 |
| 66 | Oligodendrocyte | Mobp | ENSMUSG00000032517 | 17433 |
| 67 | Oligodendrocyte | Eml1 | ENSMUSG00000058070 | 68519 |
| 68 | Oligodendrocyte | Grb14 | ENSMUSG00000026888 | 50915 |
| 69 | Oligodendrocyte | Pllp | ENSMUSG00000031775 | 67801 |
| 70 | Oligodendrocyte | Gltp | ENSMUSG00000011884 | 56356 |
| 71 | Oligodendrocyte | Slain1 | ENSMUSG00000055717 | 105439 |
| 72 | Oligodendrocyte | Cldn11 | ENSMUSG00000037625 | 18417 |
| 73 | Oligodendrocyte | Phldb1 | ENSMUSG00000048537 | 102693 |
| 74 | Oligodendrocyte | Pigz | ENSMUSG00000045625 | 239827 |
| 75 | Oligodendrocyte | Kndc1 | ENSMUSG00000066129 | 76484 |
| 76 | Oligodendrocyte | Mag | ENSMUSG00000036634 | 17136 |
| 77 | Oligodendrocyte | Pdlim2 | ENSMUSG00000022090 | 213019 |
| 78 | Oligodendrocyte | Polr3e | ENSMUSG00000030880 | 26939 |
| 79 | Oligodendrocyte | Spg20 | ENSMUSG00000036580 | 229285 |
| 80 | Oligodendrocyte | Mog | ENSMUSG00000076439 | 17441 |
| 81 | Oligodendrocyte | Nmral1 | ENSMUSG00000063445 | 67824 |
| 82 | Oligodendrocyte | Gsn | ENSMUSG00000026879 | 227753 |
| 83 | Oligodendrocyte | Carhsp1 | ENSMUSG00000008393 | 52502 |
| 84 | Oligodendrocyte | Ankrd28 | ENSMUSG00000014496 | 105522 |
| 85 | Oligodendrocyte | Lpar1 | ENSMUSG00000038668 | 14745 |
| 86 | Oligodendrocyte | Ugt8a | ENSMUSG00000032854 | 22239 |
| 87 | Oligodendrocyte | Fa2h | ENSMUSG00000033579 | 338521 |
| 88 | Oligodendrocyte | Tmem88b | ENSMUSG00000073680 | 320587 |
| 89 | Oligodendrocyte | Ermn | ENSMUSG00000026830 | 77767 |
| 90 | Oligodendrocyte | Myrf | ENSMUSG00000036098 | 225908 |
| 91 | Oligodendrocyte | Elovl7 | ENSMUSG00000021696 | 74559 |
| 92 | Oligodendrocyte | Aspa | ENSMUSG00000020774 | 11484 |
| 93 | Oligodendrocyte | Gjb1 | ENSMUSG00000047797 | 14618 |
| 94 | OPC | Evi5l | ENSMUSG00000011832 | 213027 |
| 95 | OPC | Fosb | ENSMUSG00000003545 | 14282 |
| 96 | OPC | Cdo1 | ENSMUSG00000033022 | 12583 |
| 97 | OPC | Slc35b2 | ENSMUSG00000037089 | 73836 |
| 98 | OPC | Gpt2 | ENSMUSG00000031700 | 108682 |
| 99 | OPC | Vcan | ENSMUSG00000021614 | 13003 |
| 100 | OPC | Rnf180 | ENSMUSG00000021720 | 71816 |
| 101 | OPC | Ampd3 | ENSMUSG00000005686 | 11717 |
| 102 | OPC | Sox8 | ENSMUSG00000024176 | 20681 |
| 103 | OPC | Adam9 | ENSMUSG00000031555 | 11502 |
| 104 | OPC | Cercam | ENSMUSG00000039787 | 99151 |
| 105 | OPC | Neu4 | ENSMUSG00000034000 | 241159 |
| 106 | OPC | Myt1 | ENSMUSG00000010505 | 17932 |
| 107 | OPC | Gfra1 | ENSMUSG00000025089 | 14585 |
| 108 | OPC | Chst11 | ENSMUSG00000034612 | 58250 |
| 109 | OPC | Sema3d | ENSMUSG00000040254 | 108151 |
| 110 | OPC | Galnt3 | ENSMUSG00000026994 | 14425 |
| 111 | OPC | Fam114a1 | ENSMUSG00000029185 | 68303 |
| 112 | Pyramidal | Baiap2 | ENSMUSG00000025372 | 108100 |
| 113 | Pyramidal | Slc17a7 | ENSMUSG00000070570 | 72961 |
| 114 | Pyramidal | Ptk2b | ENSMUSG00000059456 | 19229 |
| 115 | Pyramidal | Nrn1 | ENSMUSG00000039114 | 68404 |
| 116 | Pyramidal | Fhl2 | ENSMUSG00000008136 | 14200 |
| 117 | Pyramidal | Itpka | ENSMUSG00000027296 | 228550 |
| 118 | Pyramidal | Neurod6 | ENSMUSG00000037984 | 11922 |
| 119 | Pyramidal | Nptx1 | ENSMUSG00000025582 | 18164 |
| 120 | Pyramidal | Sv2b | ENSMUSG00000053025 | 64176 |
| 121 | Pyramidal | Kcnv1 | ENSMUSG00000022342 | 67498 |
| 122 | Inhibitory | Npy | ENSMUSG00000029819 | 109648 |
| 123 | Inhibitory | Gad1 | ENSMUSG00000070880 | 14415 |
| 124 | Inhibitory | Slc6a1 | ENSMUSG00000030310 | 232333 |
| 125 | Inhibitory | Gad2 | ENSMUSG00000026787 | 14417 |
| 126 | Inhibitory | Pcp4l1 | ENSMUSG00000038370 | 66425 |
| 127 | Inhibitory | Zcchc12 | ENSMUSG00000036699 | 72693 |
| 128 | Inhibitory | Dlx6os1 | ENSMUSG00000090063 | NA |
| 129 | Inhibitory | Igf1 | ENSMUSG00000020053 | 16000 |
| 130 | Inhibitory | Synpr | ENSMUSG00000056296 | 72003 |
| 131 | Ndnf_on | Fgf13 | ENSMUSG00000031137 | 14168 |
| 132 | Ndnf_on | Dner | ENSMUSG00000036766 | 227325 |
| 133 | Ndnf_on | Reln | ENSMUSG00000042453 | 19699 |
| 134 | Ndnf_on | Pnoc | ENSMUSG00000045731 | 18155 |
| 135 | Ndnf_on | Adarb2 | ENSMUSG00000052551 | 94191 |
| 136 | Ndnf_on | Kit | ENSMUSG00000005672 | 16590 |
| 137 | Ndnf_on | Cnr1 | ENSMUSG00000044288 | 12801 |
| 138 | Ndnf_on | Parm1 | ENSMUSG00000034981 | 231440 |
| 139 | Ndnf_on | Klhl13 | ENSMUSG00000036782 | 67455 |
| 140 | Ndnf_on | Nr2f2 | ENSMUSG00000030551 | 11819 |
| 141 | Ndnf_on | Adra1a | ENSMUSG00000045875 | 11549 |
| 142 | Ndnf_on | Hapln1 | ENSMUSG00000021613 | 12950 |
| 143 | Ndnf_on | Hdac9 | ENSMUSG00000004698 | 79221 |
| 144 | Sncg_on | Sncg | ENSMUSG00000023064 | 20618 |
| 145 | Sncg_on | Nrip3 | ENSMUSG00000034825 | 78593 |
| 146 | Sncg_on | Fxyd6 | ENSMUSG00000066705 | 59095 |
| 147 | Sncg_on | Kcnip1 | ENSMUSG00000053519 | 70357 |
| 148 | Sncg_on | Sema3c | ENSMUSG00000028780 | 20348 |
| 149 | Sncg_on | Dlx1 | ENSMUSG00000041911 | 13390 |
| 150 | Sncg_on | Maf | ENSMUSG00000055435 | 17132 |
| 151 | Sncg_on | Rgs12 | ENSMUSG00000029101 | 71729 |
| 152 | Sncg_on | Htr3a | ENSMUSG00000032269 | 15561 |
| 153 | Sncg_on | Fstl5 | ENSMUSG00000034098 | 213262 |
| 154 | Sncg_on | Gng4 | ENSMUSG00000021303 | 14706 |
| 155 | Sncg_on | Qpct | ENSMUSG00000024084 | 70536 |
| 156 | Sncg_on | Galnt14 | ENSMUSG00000024064 | 71685 |
| 157 | Sncg_on | Sp8 | ENSMUSG00000048562 | 320145 |
| 158 | Sncg_on | Cxcl14 | ENSMUSG00000021508 | 57266 |
| 159 | Pvalb_on | Pvalb | ENSMUSG00000005716 | 19293 |
| 160 | Pvalb_on | Nxph1 | ENSMUSG00000046178 | 18231 |
| 161 | Pvalb_on | Ubash3b | ENSMUSG00000032020 | 72828 |
| 162 | Pvalb_on | Nek7 | ENSMUSG00000026393 | 59125 |
| 163 | Pvalb_on | Gm13629 | ENSMUSG00000087301 | NA |
| 164 | Pvalb_on | Kcnc2 | ENSMUSG00000035681 | 268345 |
| 165 | Pvalb_on | Cox6a2 | ENSMUSG00000030785 | 12862 |
| 166 | Pyramidal_on | Nrgn | ENSMUSG00000053310 | 64011 |
| 167 | Pyramidal_on | Arpp21 | ENSMUSG00000032503 | 74100 |
| 168 | Pyramidal_on | Rgs4 | ENSMUSG00000038530 | 19736 |
| 169 | Pyramidal_on | Ctxn1 | ENSMUSG00000048644 | 330695 |
| 170 | Pyramidal_on | Stx1a | ENSMUSG00000007207 | 20907 |
| 171 | Pyramidal_on | Rasgrp1 | ENSMUSG00000027347 | 19419 |
| 172 | Pyramidal_on | Dkk3 | ENSMUSG00000030772 | 50781 |

Supplementary Table 4: List of cell type-specific markers based on re-analysis of published dissociated cell-based scRNAseq experiments from mouse brain.
